# Genome-wide core sets of SNP markers and Fluidigm assays for rapid and effective genotypic identification of Korean cultivars of lettuce (*Lactuca sativa* L.)

**DOI:** 10.1093/hr/uhac119

**Published:** 2022-05-26

**Authors:** Jee-Soo Park, Min-Young Kang, Eun-Jo Shim, JongHee Oh, Kyoung-In Seo, Kyung Seok Kim, Sung-Chur Sim, Sang-Min Chung, Younghoon Park, Gung Pyo Lee, Won-Sik Lee, Minkyung Kim, Jin-Kee Jung

**Affiliations:** Seed Testing and Research Center, Korea Seed & Variety Service, Gimcheon 39660, Republic of Korea; Seed Testing and Research Center, Korea Seed & Variety Service, Gimcheon 39660, Republic of Korea; Seed Testing and Research Center, Korea Seed & Variety Service, Gimcheon 39660, Republic of Korea; Seed Testing and Research Center, Korea Seed & Variety Service, Gimcheon 39660, Republic of Korea; Seed Testing and Research Center, Korea Seed & Variety Service, Gimcheon 39660, Republic of Korea; Department of Natural Resource Ecology and Management, Iowa State University, Ames IA 50011, USA; Department of Bioresources Engineering, Sejong University, Seoul 05006, Republic of Korea; Department of Life Sciences, Dongguk University, Seoul 04620, Republic of Korea; Department of Horticultural Bioscience, Pusan National University, Miryang 50463, South Korea; Department of Plant Science and Technology, Chung-Ang University, Ansung 17546, South Korea; Seed Testing and Research Center, Korea Seed & Variety Service, Gimcheon 39660, Republic of Korea; Department of Bioresources Engineering, Sejong University, Seoul 05006, Republic of Korea; Seed Testing and Research Center, Korea Seed & Variety Service, Gimcheon 39660, Republic of Korea

## Abstract

Lettuce is one of the economically important leaf vegetables and is cultivated mainly in temperate climate areas. Cultivar identification based on the distinctness, uniformity, and stability (DUS) test is a prerequisite for new cultivar registration. However, DUS testing based on morphological features is time-consuming, labor-intensive, and costly, and can also be influenced by environmental factors. Thus, molecular markers have also been used for the identification of genetic diversity as an effective, accurate, and stable method. Currently, genome-wide single nucleotide polymorphisms (SNPs) using next-generation sequencing technology are commonly applied in genetic research on diverse plant species. This study aimed to establish an effective and high-throughput cultivar identification system for lettuce using core sets of SNP markers developed by genotyping by sequencing (GBS). GBS identified 17 877 high-quality SNPs for 90 commercial lettuce cultivars. Genetic differentiation analyses based on the selected SNPs classified the lettuce cultivars into three main groups. Core sets of 192, 96, 48, and 24 markers were further selected and validated using the Fluidigm platform. Phylogenetic analyses based on all core sets of SNPs successfully discriminated individual cultivars that have been currently recognized. These core sets of SNP markers will support the construction of a DNA database of lettuce that can be useful for cultivar identification and purity testing, as well as DUS testing in the plant variety protection system. Additionally, this work will facilitate genetic research to improve breeding in lettuce.

## Introduction

Lettuce (*L. sativa* L., 2n = 2x = 18) is one of the agriculturally important leaf vegetables belonging to the Asteraceae family and is cultivated mainly in temperate climate areas of the world. In Korea, the production of lettuce was estimated to be over 93 543 tons from 3773 ha in 2018 [1]. The genome of lettuce has been completely decoded [2], and modeling analysis of approximately 45 000 genes has been conducted. New cultivars of lettuce are developed and commercialized every year, and identification of each cultivar is important for the registration and protection measures of newer cultivars. However, the identification of lettuce cultivars is a difficult task due to their close genetic relationships. Lettuce is a morphologically diverse crop and can be classified into different horticultural types based on head and leaf shape, size, structure, and stem length as well as end uses [3]. According to the International Union for the Protection of New Varieties of Plants (UPOV) TG/13/10 guidelines for lettuce [4], lettuce can be grouped into diverse types, such as butterhead, Iceberg, Frisée d’Amerique (loose-leaf), Oakleaf, and Cos (Romaine).

The UPOV defines the rights of plant breeders and protects them from unauthorized utilization of new cultivars. For cultivar registration and protection, distinctness, uniformity, and stability (DUS) testing in a UPOV system of plant variety protection (PVP) is required. However, DUS testing, which is based on morphological features, is costly, time-consuming, and labor-intensive. Furthermore, it can be influenced by various environmental conditions that can impose limitations on the identification of cultivars [5]. Therefore, DUS testing needs to be supported by genetic analysis using molecular markers. The UPOV has agreed to the deployment of molecular markers for the identification of cultivars that are specifically linked to a phenotypic trait [6]. In addition, the working group on Biochemical and Molecular Techniques and DNA-profiling in Particular (BMT), a technical committee under the UPOV, has discussed the usage of molecular markers for the identification and protection of cultivars [6, 7]. Diverse molecular markers, such as simple sequence repeats (SSRs) and single nucleotide polymorphisms (SNPs), have been applied in various plants for the identification and purity assessment of cultivars [8–16].

Before registration of a new cultivar, DUS tests are carried out to determine whether a new cultivar is distinct, uniform and stable. As a part of DUS testing, the distinctness of the new cultivar is examined by comparison with a similar existing cultivar, which is called “reference varietiy”. SNP markers have been routinely utilized as a tool for the management of reference varieties for DUS examinations. Among the grouped cultivars, the one with the highest genetic similarity to the unknown cultivar can be selected as a “similar variety” and used for the DUS test. In other words, more relevant reference varieties can be selected for DUS testing based on their DNA profiles, and the duration and cost of DUS testing can be reduced.

SSR markers have been widely used for the assessment of phylogenetic relationships and DUS testing in commercial lettuce cultivars [10, 17–23] because of their advantages of being co-dominant and multi-allelic [24, 25] (Choi et al., 2016; Kong et al., 2020). Hong et al. [26] constructed expressed sequence tag-SSR profiles to identify 92 lettuce cultivars from Korea and proposed the utility of the markers in the distinctiveness tests of lettuce. Zhou et al. [8] developed a set of 19 SSR markers for the identification of 73 cultivars of head lettuce (*Lactuca L. sativa capitate* L.). SSR markers were also used to characterize the genetic diversity of the germplasm of chicory (*Cichorium intybus*), which also belongs to the same family, Asteraceae, as lettuce [27]. However, SSR markers have limitations since they are less reproducible and time-consuming and expensive to develop [28].

Compared to SSR markers, SNP markers are bi-allelic, making it simple to merge data between groups, and it is possible to generate large databases of marker information coupled with high-throughput genotyping. Currently, SNPs are the most preferred in cultivar identification and genomic studies due to their high abundance, stability, and efficiency [29, 30]. For example, a large collection of SNPs was identified from 223 pumpkin cultivars via genotyping by sequencing (GBS), and core markers were selected for cultivar identification in pumpkin [14]. Phylogenetic studies, evaluation of genetic variation and population structure, genome-wide association studies, and construction of genetic linkage maps based on lettuce SNP markers and genotype data have been applied to facilitate efficient genetic studies in lettuce [3, 31–34]. Truong et al. [31] constructed the linkage map of lettuce using 1113 SNPs via sequence-based genotyping. The genetic diversity and population structure were investigated using SNP markers from 380 lettuce accessions, which were maintained by the United States Department of Agriculture [3, 32]. The genotype data have been successfully used to identify lettuce cultivars, indicating that SNP markers can be useful for the rapid evaluation of genetic variation and population structure in the lettuce germplasm collection [18, 59]. In addition, research on genome-wide marker-trait association in lettuce has been conducted [32–35, 62, 63]. However, cultivar identification of commercial lettuce using SNP markers in Korea is still in its infancy.

Recent advancement of next-generation sequencing (NGS) technologies has enabled researchers to analyze and utilize genetic resources efficiently [36, 37, 60]. NGS technology has also accelerated high-throughput and genome-wide SNP genotyping [36–40]. GBS, one of the widely used NGS methods, is a high-throughput and cost-effective approach for discovering genome-wide SNPs. GBS has been used for the examination of genetic diversity in various plants, and SNP data from GBS have been applied for diverse genetic studies, cultivar identification, and marker-assisted breeding (MAB) [13–16, 33, 37–47, 61]. In lettuce, GBS has been successfully used to provide a large number of highly informative genome-wide SNPs [33].

Currently, diverse automated platforms for high-throughput analysis have enabled the analysis of large amounts of data within a short period [48, 49]. For example, Fluidigm dynamic arrays adopt an automated PCR and a nanofluidic integrated fluid circuit (IFC) [50]. SNP genotyping and the development of SNP markers for cultivar identification using the Fluidigm platform are being widely used for various plants [13–16, 51–54]. However, SNP markers for identifying different cultivars of commercial lettuce in Korea have not been sufficiently developed.

In this study, we developed core sets of genome-wide SNP markers to identify cultivars of lettuce using the GBS and SNP-genotyping approach. We validated these core sets of SNPs to develop molecular markers for high-throughput analysis using the Fluidigm platform. We also evaluated the utility of core SNPs using genetic differentiation analysis. These developed SNP markers will be useful for database construction and will facilitate cultivar identification, purity testing, and breeding of lettuce.

## Results

### Genome-wide SNP discovery in commercial lettuce cultivars

Using the GBS approach, a total of 549 123 132 reads were generated with an average of 6 034 320 reads per individual cultivar from the 90 Korean commercial lettuce cultivars analyzed. After barcode and adapter sequences were trimmed and low-quality reads were filtered, 443 005 356 clean reads were obtained (Table 1). About 86% of the reads were successfully mapped to the *L. sativa* cv. *Salinas* (v8) reference genome, with an average read depth of 19X [55].

**Table 1:** Summary of GBS data for 90 lettuce cultivars

Class	No.
Total number of raw reads	549 123 132
Average number of raw reads per cultivar	6 034 320
Total length of raw reads	55 461 436 332
Total number of trimmed reads	443 005 356
Total number of mapped reads	380 064 388
Total SNP	276 462
Filtered SNP	17 877

A total of 276 462 SNPs were identified through genome-wide SNP identification. Among the SNPs identified from 90 cultivars used in this study, transition (A/G or C/T) and transversion (A/C, A/T, C/G or G/T) SNPs accounted for 62.8% and 37.2%, respectively, with a transitions-to-transversions ratio of 1.69. Both types of transition SNPs (C/T and A/G) were detected in similar numbers. Among transversion SNPs, the A/T type showed higher numbers than other types (Table S1).

**Figure 1:**
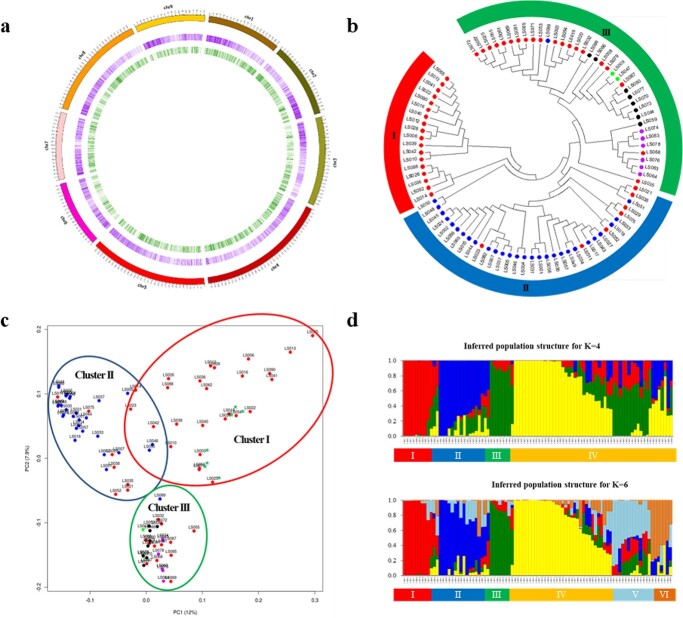
Chromosomal distribution and genetic diversity analyses of 17,877 SNP loci from 90 lettuce cultivars. a Distribution of genes and 17,877 SNP loci in lettuce genome. The middle purple circle illustrates gene distribution and the innermost green circle illustrates SNP distribution per 500kb. b Neighbor-joining phylogenetic tree using 17,877 SNPs in 90 lettuce cultivars. c Principal component analysis of the 90 lettuce cultivars genotyped with 17,877 SNPs. d Population structure of the 90 lettuce cultivars using 17,877 SNPs based on the STRUCTURE output for K=4 and K=6. The x axis shows the different lettuce cultivar and y axis represents co-ancestary coefficient. In b, c, colors of dots reflect the morphological types of lettuce; Cos (red), Iceberg (green), Frisée d'Amérique (blue), Butterhead (purple), Lollo, Multi-divided, and Oakleaf type (black). Differently colored outlines or bars indicate the main clusters.

Low-quality SNPs and with significant polymorphism among the 90 cultivars were filtered out. After filtering, 17 877 high-quality SNPs (minor allele frequency (MAF) > 0.05; missing data < 30%) were identified. The chromosomal distribution of these 17 877 SNP loci and genes in the lettuce genome is depicted in Fig. 1a. Generally, the SNPs were evenly distributed across the chromosomes.

Out of the 17 877 SNPs identified, 12 959 SNPs (72.5%) were located in intergenic regions and 4928 (27.6%) in genic regions, of which 3502 (19.6%) were located in exons and 1416 (7.9%) were in introns (Table 2). From the 3279 filtered SNPs derived from coding sequences, 2139 (65.2%) were found to be synonymous SNPs that do not alter the amino acid sequences of the polypeptide, whereas 1140 (34.8%) were discovered to be non-synonymous SNPs that cause changes to the amino acid sequences (Table 2). Of the non-coding sequence SNPs, 689 (3.9%) were derived from upstream and downstream regions of the genes.

**Table 2:** The chromosomal location of 17 877 SNPs identified in 90 lettuce cultivars

Class	Total SNPs	Candidate SNPs	Core SNPs
Genic region	4928 (27.6%)	222 (75.5%)	159 (82.8%)
CDS - Synonymous	2139	133	96
CDS - Non-synonymous	1140	60	43
UTR	223	16	10
Intron	1416	13	10
Intergenic region	12 959 (72.5%)	72 (24.5%)	33 (17.2%)
Intergenic	12 270	67	31
Upstream gene	376	3	2
Downstream gene	291	2	0
Up/Downstream gene	22	0	0
Total number of SNPs	17 877	294	192

Furthermore, transition (A/G or C/T) and transversion (A/C, A/T, C/G or G/T) SNPs accounted for 68.5% and 31.5%, respectively, with a transitions-to-transversions ratio of 2.18 (Table S1). Both types of transition SNPs (A/G and C/T) were detected in similar numbers. Among transversion SNPs, the A/C type showed higher numbers than other types.

### Genetic diversity within lettuce cultivars

The level of genetic diversity, the polymorphic information content (PIC) values, MAF, and the heterozygosity of the 17 877 SNPs filtered from 90 lettuce cultivars were calculated (Table S2). As a result, PIC values ranged from 0.10 to 0.38, with an average PIC of 0.27. MAF for the selected SNP markers was from 0.06 to 0.50, with an average of 0.24, and the heterozygosity ranged from 0.10 to 0.38, with an average of 0.27. The selected 17 877 SNPs were used for the genetic analyses, including the phylogenetic analysis, principal component analysis (PCA), and population structure analysis.

A phylogenetic tree was constructed using the neighbor-joining method. The result showed that the 90 cultivars were classified into three divergent groups (Fig. 1b): 18 cultivars were classified into Cluster I (Red), 35 cultivars into Cluster II (Blue), and the 37 cultivars into Cluster III (Green). Generally, lettuce cultivars were clustered according to their horticultural types. Cluster I only comprised cultivars of the Cos (Romaine) type, and 27 out of 28 lettuce cultivars of Frisée d’Amerique type as well as several cultivars of the Cos type were clustered into Cluster II. In Cluster III, cultivars of the Cos, butterhead and Iceberg types were clustered, and there was a tendency to form subgroups by the horticultural types.

After conducting PCA using the 17 877 SNPs to investigate the genomic differences of lettuce cultivars, the 90 lettuce cultivars were classified into three groups (Fig. 1c). The top two principal components (PC1 and PC2) accounted for 19.9% of the genetic variation among the 90 cultivars. In addition, PC3 explained 4.4% of the observed variances (Data not shown). With a few exceptions, the phylogenetic relationships of different lettuce groups were in good agreement with the PCA results (Fig. 1b, c), resulting in three major clusters for the 90 cultivars, and each cluster generally comprised the same horticultural type. The majority of the cultivars in Cluster I was Cos type, while Cluster II contained 27 of the 28 Frisée d’Amerique type accessions. Cluster III included all six butterhead type accessions and two Iceberg type accessions, as well as several Cos type accessions. Seven accessions (LS003, LS019, LS020, LS025, LS053, LS054, and LS071) showed different grouping in phylogenetic (Cluster III) and PCA (Cluster I) analyses.

The population structure analysis for the 90 lettuce cultivars using the 17 877 filtered SNPs determined the optimal number of populations (K = 4) corresponding to the highest peak in the Delta-K graph (Fig. 1d and Fig. S1). The result suggested that genetic variations in the 90 cultivars can be divided into four major clusters, which was similar to the results of the PCA and phylogenetic analysis. Cluster I was composed of a mixture of 12 lettuce cultivars consisting of the Cos, Iceberg, multi-divided type, and unknown morphological type. Cluster II consisted of 17 lettuce cultivars, all of which were of the Cos type. All six Butterhead-type accessions, one Cos type accession, and one Lollo type accession were in Cluster III. All accessions of Frisée d’Amerique type were in Cluster IV. Most of the Cos-type accessions were found in two clusters, Cluster II (17 Cos-type) and Cluster IV (23 Cos-type), whereas four Cos-type accessions were found in Cluster I and one in Cluster III.

The second-highest peak in the Delta-K graph was found when K = 6, assuming six subgroups among the 90 cultivars (Fig. 1d and Fig. S1). No dramatic changes in clustering were observed when K was increased from 4 to 6, except for Cluster IV, with 29 Frisée d’Amerique type, 23 Cos-type, and one Oakleaf-type accessions being divided into three subgroups. Specifically, subgroup 1 of Cluster IV consisted of a majority of Frisée d’Amerique type lettuce accessions. Cos-type accessions were spread among all three subgroups: five in subgroup 1, 11 in subgroup 2, and seven in subgroup 3. Lettuce cultivars in subgroup 3 were composed of Cos-type accessions only.

Generally, cultivars with identical morphological types were grouped in the same cluster and a substantially close association was observed between SNP genotypes and horticultural types. In addition, the clusters obtained from the phylogenetic tree using the NJ method were in an agreement with those from STRUCTURE and PCA.

### Development and validation of the core SNP assays for lettuce cultivar identification

To develop SNP markers that are effective for cultivar identification, we selected 294 SNP markers with a PIC value higher than 0.1 and with significant polymorphism between the 90 lettuce cultivars. Of these selected SNPs, 226 SNPs were in genic regions of the lettuce genome. To validate the selected SNPs, the sequence differences between cultivars were confirmed by Sanger sequencing (Data not shown). Subsequently, primer sets for the Fluidigm assay were designed for each confirmed SNP. Fluidigm-based genotyping was conducted for the 90 lettuce cultivars used for GBS and the additional five commercial cultivars from the Netherlands. Genotype calls from the SNP assay for 95 lettuce cultivars are shown in the scatter plot (Fig. S2). Homozygous types, XX and YY, were labeled with fluorescent dyes, FAM or HEX, respectively, represented by red and green points. Heterozygous marker type (XY) was labeled with both fluorescent dyes FAM and HEX, represented by blue points. Among 294 SNP markers, SNPs which showed clear separation between two homozygous genotypes were selected for the development of the core marker sets (Fig S2a, b), and SNPs with unusual clustering patterns including heterozygous genotypes were filtered out (Fig. S2c). The automatically-called heterozygous genotypes by the software, as shown in Fig. S2b, were manually changed to homozygote genotypes.

**Figure 2:**
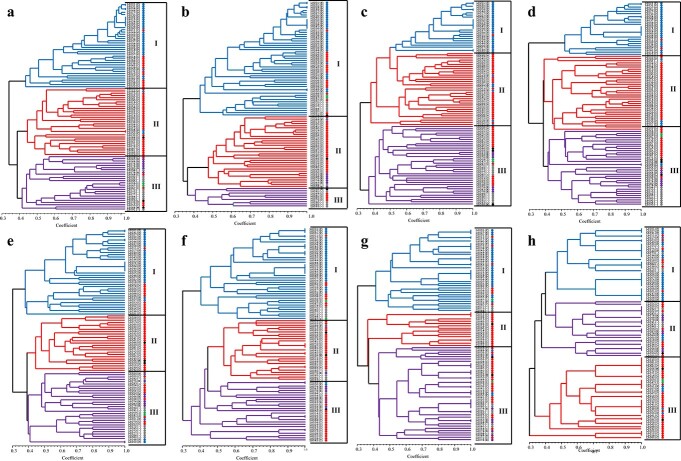
Phylogenetic trees of the 95 commercial lettuce accessions using the subsets of 192 (a), 96 (b), 48 (c), 24 (d), 18 (e), 12 (f), 9(g), and 6 (h) markers. The color of dot indicates horticultural type of each accession. The color of present Similarity coefficients are presented at the bottom of the trees.

**Figure 3:**
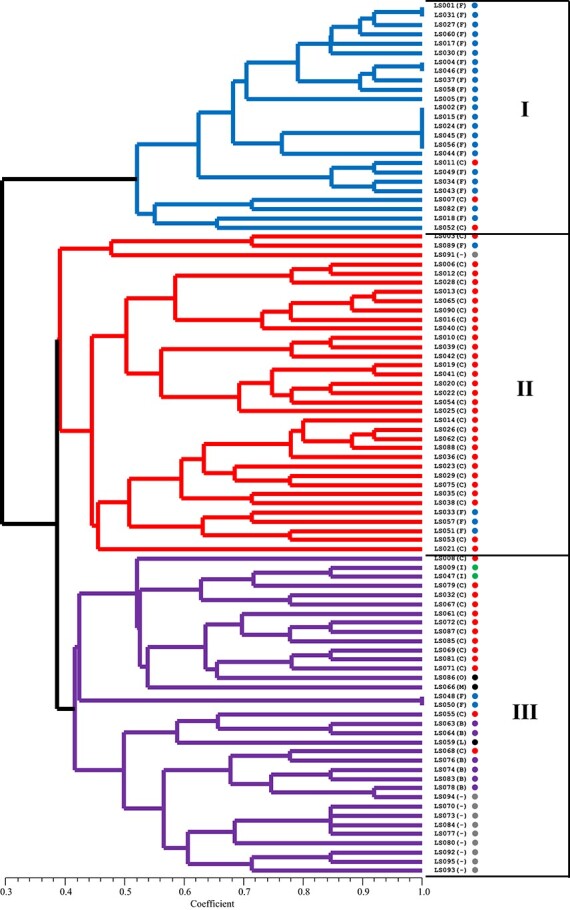
The phylogenetic tree of 95 commercial cultivars using 24 core SNP markers. The color of circle next to names of varieties indicate horticultural types; Cos type (red), Iceberg type (green), Frisée d'Amérique type (blue), Butterhead type (purple), unknown (gray), other types including Lollo, Multi-divided, and Oakleaf type (black). All three distinct groups were assigned by different colors. Jaccard’s similarity coefficients are presented at the bottom of the trees.

The 294 filtered markers were effective in distinguishing 84 (88.4%) of the 95 lettuce cultivars. Besides, the samples which were not separated by the 294 markers could be used as a “reference variety” for each matching sample in DUS testing. To develop the core markers for cultivar identification via the Fluidigm system, sets of core markers were selected based on polymorphisms from the lettuce SNP data mined from GBS considering high polymorphism based on PIC value (Table S2). The average PIC value of the 192 core markers was 0.32, ranging from 0.1 to 0.38. The PIC values of the 96 core markers ranged from 0.11 to 0.38, with an average of 0.31. Similarly, the PIC value of the 48 core markers ranged from 0.11 to 0.38, with an average of 0.33. The average PIC value of the 24 core markers was 0.33, ranging from 0.23 to 0.37. These SNP sets are suitable for high-throughput systems such as the IFC platforms of Fluidigm genotyping assays. The selected SNP markers and their related gene annotations are summarized in Table S2.

To evaluate the performance for cultivar identification, a phylogenetic tree was constructed using the selected 192 SNP markers based on the genotyping results (Fig. 2a, Table S3). The 192 markers identified 84 (88.4%) of the 95 lettuce cultivars, like the 294 markers mentioned above, and were separated into three main clusters. The Frisée d’Amérique type was found almost exclusively in Cluster I, except for three cultivars that were grouped into other clusters. The cultivars of the Cos type were included in all clusters but were found mainly clustered in Cluster II. The Oakleaf types tended to be clustered with the Cos types, and the rest of the types tended to be clustered with each other.

Among the 192 SNP markers, the 96 core SNPs that showed significant polymorphism among the 95 cultivars were selected for further lettuce cultivar identification (Fig. 2b). Genetic differentiation using the 96 core SNPs was compared with those of distinct SNPs derived from GBS. As shown in Fig. 2b, the phylogenetic tree displayed three main clusters that are assigned different colors. The result showed that the accessions of identical horticultural types were included in the same cluster. All Frisée d’Amérique-type accessions were included in Cluster I, mainly in the subgroup Cluster I-1. Whereas, Cos-type accessions were spread into every subgroup: 17 in Cluster I, 24 in Cluster II, and four in Cluster III. Cluster I-4 comprised all accession types including Iceberg, Lollo, and multi-divided types, and six accessions of an unknown type. Cluster II included 19 Cos-type accessions and one Oakleaf type. Notably, Cluster II-2 contained all Butterhead-type accessions, and the accessions of an unknown type were clustered in Cluster I-4 and III. Representatively, the number of subgroups of 95 lettuce accessions was estimated using 96 core SNP markers (Fig. S3). The population structure analysis based on the genotyping results of 96 core SNPs indicated that the optimal number of subpopulations was three or seven, and this result was consistent with the phylogenetic tree constructed with 96 SNP markers.

From the 96 core markers, 48 and 24 markers were selected and phylogenetic analysis was performed using these markers. The 48 and 24 markers also identified 84 (88.4%) of the 95 lettuce cultivars, like the 294 markers (Fig. 2c, d). The 95 cultivars were separated into three main clusters, and associations between SNP genotypes and horticultural types were also detected (Fig. 3). Therefore, a set of 24 SNP markers would be sufficient for the selection of the reference varieties for the DUS examination, which would support UPOV-based PVP system. The genotyping results and the primer sequences of the 24 core SNP markers developed in this study have been provided in Table 3 and 4.

Additionally, a smaller number of markers were selected and analyzed to calculate the minimal number of SNP markers that can distinguish all tested cultivars. Eighteen markers were able to differentiate 84 (88.4%) of the 95 lettuce cultivars, similar to the 294 markers, while 12 markers were able to identify 67 (70.5%) of the 95 cultivars (Fig. 2e, f). Nine and six SNP markers detected genetic variations to distinguish 48 (50.5%) and 16 (16.8%) cultivars, respectively (Fig. 2 g, h).

**Figure 4:**
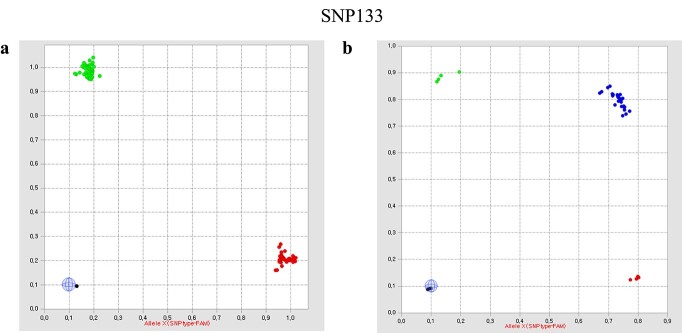
Representative clustering patterns of homozygotes and the artificial heterozygotes. Colored dots presents the genotypes from the same SNP marker of the 95 lettuce cultivars (a) and DNA mixtures from two cultivars with different alleles (b). Each color code in the plots presents one of three genotypes: homozygote of allele 1 (red), homozygote of allele 2 (green), and heterozygote (blue).

To validate the developed 24 core markers, Fluidigm-based genotyping was performed for the sets of DNA mixtures and original samples and their genotyping results were compared. Fig. 4 shows the genotyping results of the original samples and their mixture. Original data showed two clusters corresponding to two homozygous genotypes (XX, red; YY, green) (Fig. 4a). However, heterozygous genotypes (XY, blue) were identified from some DNA mixtures (Fig. 4b). Among the reactions in which the genotypes of the two samples were different from each other, 93.3% genotypes of the mixture were heterozygous. Thus, we have determined that the developed markers could identify not only homozygous lines but also heterozygous lines.

## Discussion

The main goal of this study was to develop a core set of SNP markers suitable for the Fluidigm assay to create a fast and high-throughput screening system for the identification of lettuce cultivars. In this study, the selected 17 877 SNPs using the GBS approach could successfully differentiate 90 commercial lettuce cultivars in Korea. A similar classification pattern was observed with phylogenetic analysis and PCA where both analyses classified the 90 lettuce cultivars into three distinct groups (Fig. 1). In both analyses, most of the cultivars included in Cluster I were Cos type, while Cluster II included the Frisée d’Amerique type as the majority. In Cluster III, Cos, Butterhead, and Iceberg types were present, and subgroups were formed within a horticultural type. Population structure analysis identified four genetic clusters, which nearly corresponded to the grouping of the accessions from phylogenetic and PCA analyses. Lettuce cultivars were generally classified into three main clusters according to their horticultural types. Therefore, these results demonstrate that the SNPs can be variable resources to reveal the association between genomic variations and horticultural types of lettuce.

**Table 3:** The genotypes of 24 core SNP markers in 95 commercial lettuce varieties

${\includegraphics{\bwartpath uhac119t3a}} $
${\includegraphics{\bwartpath uhac119t3b}} $

**Table 4:** Summary of the information of 24 core SNP markers developed in this study

**No.**	**Chromosome**	**Position**	**Allele**	**SNP location**	**giNumber**	**MAF** ^**a**^	**PIC** ^**b**^	**ASP1 sequence**	**ASP2 sequence**	**LSP sequence**	**STA sequence**
SNP001	chr1	11 945 871	A/G	CDS	gi|357 464 531	0.49	0.37	CTCTTCGCCAGTGCCACT	TCTTCGCCAGTGCCACC	GTCAATGACGCCGGCGTT	CTTTGTCTGGGAGCTGCAC
SNP002	chr1	13 440 345	G/C	CDS	gi|1 137 180 833	0.48	0.37	AGGAGTATTAACAGCAGCTGTCC	AGGAGTATTAACAGCAGCTGTCG	CGGAACGTTGCCACTCGC	CCGATTGGAAACGACGGC
SNP005	chr1	44 033 573	C/T	Intron	gi|976 911 262	0.22	0.28	TGGCAGGTAACTAACTAACTGACTG	GTGGCAGGTAACTAACTAACTGACTA	ACGCCAATCCCCTGTATCACA	GCTTCTTTGGTGTCAATGTTGGT
SNP010	chr1	69 220 682	C/A	CDS	gi|976 926 764	0.26	0.31	CCTCCCATGAACAACAATCAAAACTAG	CCTCCCATGAACAACAATCAAAACTAT	GCTGCTCATGGCTCTCAAGTG	TGCTGCATGCAACCCC
SNP025	chr2	91 614 666	T/C	CDS	gi|976 924 026	0.22	0.28	CTTCTGATGCATGAAGACCATGT	CTTCTGATGCATGAAGACCATGC	GCAATCAGAAACTCTGAAAATGATGATTGGA	GTTGTTGTAAAACTTGCACTGCT
SNP038	chr2	117 891 323	C/T	Intergenic	-	0.44	0.37	ACCAAGGAACTAGAGGACGAAATC	AACCAAGGAACTAGAGGACGAAATT	CTGCATTCTCTTTCATTGCTTCCCT	AACTAATTGCGGGTCTTAATGCTAA
SNP057	chr3	68 819 733	G/T	CDS	gi|976 908 992	0.42	0.37	CATCATATACAGGTAGGGTGCACC	CATCATATACAGGTAGGGTGCACA	CCTGTACTTGCAGCCCGGT	CTTTCTCTGATTTGATAATTGTTTGATGTTCA
SNP063	chr3	144 267 704	A/G	Intergenic	-	0.21	0.28	GTCCACAACGTTTTTGGGGT	TCCACAACGTTTTTGGGGC	CCCTAATAGCCTACATGTATATAAAGGACCC	ACGTGTGAAAACCCTAAAGAACAT
SNP079	chr4	226 988 250	G/A	CDS	gi|1 098 831 891	0.49	0.37	CTACGAAAATAGAAAGATCAGAAGAAGCG	CCTACGAAAATAGAAAGATCAGAAGAAGCA	TGGGCTACATTTAGCACGGGA	CGTCAGTTACATCTGCAGCTA
SNP082	chr5	8 785 574	A/G	Intergenic	-	0.16	0.23	CACCCGCTACCCGATCA	CACCCGCTACCCGATCG	GGCCGCACTTATGGGAAGTT	CCTTGCGAAACTTGTGGCA
SNP084	chr5	36 437 007	C/T	Intron	gi|976 912 394	0.43	0.37	TGCCATCAGCTACAGTGAGTAATC	TGCCATCAGCTACAGTGAGTAATT	CTCTTGACTATCCTTTCAATTCATTAATACGAAACAG	CGTCTGACTTTAGCTGCAATGATTA
SNP120	chr5	284 742 114	C/T	CDS	gi|976 892 502	0.32	0.34	ATCAGCGGAACCCCCG	CATCAGCGGAACCCCCA	CCAGAGGGGATTGCTGCAT	TCCATAGGTCCTCACTTGTGC
SNP133	chr7	112 702 864	T/G	CDS	gi|976 916 844	0.39	0.36	CTCACCCTTTGTTTGAGCCAT	CTCACCCTTTGTTTGAGCCAG	GCAGCTTGTCCAAAGAGCCA	CTGCTCGGTTCTTTGATACCC
SNP135	chr7	133 109 575	C/A	UTR	gi|1 130 837 053	0.42	0.37	TTCTCAGCCTCGATTCCGG	GTTCTCAGCCTCGATTCCGT	GCTTCTTCTCCATCTCCGCCA	GGCGGAGATGACCCGT
SNP139	chr8	32 465 725	G/A	CDS	gi|976 920 382	0.45	0.37	AATGAAACGATTTGATTAGCTTCTCAAGTATC	AATGAAACGATTTGATTAGCTTCTCAAGTATT	TGGCAAGTCTATGAAGCAGCCT	TGCAGTTCACGAGGAGGT
SNP140	chr8	32 465 762	A/G	CDS	gi|976 920 382	0.45	0.37	CTGCAGTTCACGAGGAGGTAT	CTGCAGTTCACGAGGAGGTAC	GGGATACTTGAGAAGCTAATCAAATCGTTTC	TGAAGGACATATTTAGGATCTTTGTGC
SNP141	chr8	32 467 169	G/A	CDS	gi|976 920 382	0.18	0.26	CTCAATCAGTGATTGGTTCTCGATTTC	CTCAATCAGTGATTGGTTCTCGATTTT	GGCTTCACAGAGGCTTCTAATTTTGATG	GCTTCTCAGACTCTTTGCTTCC
SNP147	chr8	76 000 585	C/T	CDS	gi|976 929 628	0.4	0.37	ACAGAAGGAGGACGAAACAACG	ACAGAAGGAGGACGAAACAACA	TCGCAGCTTCGGATTCCTCT	GTCGAAGAAGAAGAAGAACAAACAGA
SNP148	chr8	78 404 531	C/T	CDS	gi|731 395 356	0.32	0.34	AACGTCATGTCCAGACGACTC	AAACGTCATGTCCAGACGACTT	CAGCAAATCGGTTGGTTGCAG	CAGCTTTTGTTGCTCCAATAACAA
SNP158	chr8	208 004 895	C/T	CDS	gi|976 919 073	0.25	0.31	TTTCATGCTTTTTAGGCTTCCCC	GTTTCATGCTTTTTAGGCTTCCCT	TGCCGGATCACCGAAGCTTA	CAGCCATGGATATGGATCCATT
SNP166	chr9	32 125 232	A/G	CDS	gi|976 901 682	0.36	0.36	GGCTGCTAGATTTCATTCTCACTGT	GCTGCTAGATTTCATTCTCACTGC	GGATGGTAGTACAACCGGGCA	GGGAAATACGTTGAGATACTGGATT
SNP177	chr9	77 623 861	G/A	CDS	gi|976 926 002	0.36	0.36	GAGTCAACTGTTTCCTGGTGTTC	TGAGTCAACTGTTTCCTGGTGTTT	CCGGTGCCACGTGTCC	TGATTAGCGCATTCACGTTTGT
SNP180	chr9	133 535 460	G/A	CDS	gi|976 909 018	0.26	0.31	AGCTGAAGCAGCGGTGG	AGCTGAAGCAGCGGTGA	GCCGCGCTCCGCCTA	AACGATAAATCGGGAGTGATAGAGA
SNP181	chr9	135 041 719	C/T	CDS	gi|976 927 416	0.26	0.31	AGGTGATCAAGAAGCTGGATGAC	AAGGTGATCAAGAAGCTGGATGAT	CCCAGGGTCAGGCTTTCG	ACTTAACCAAAGAAAAGAAGGTGATCA

a minor allele frequency

b polymorphic information content

Core markers for Fluidigm SNP genotyping were selected based on the PIC values, which provide information on the extent of polymorphism revealed by the DNA marker and supports estimating relationships between cultivars [64, 65]. In general, the PIC value of multi-allelic markers, such as RAPD [56], AFLP [57, 58], and SSR markers can be as high as 0.5–1.0, while the PIC values of bi-allelic SNP markers range from 0–0.5 [66–68]. The comparatively high PIC value (mean PIC = 0.33) for the present core marker sets in this study will warrant that their classification accuracy. A subset of 294 SNPs selected from the 17 877 SNPs showed the genetic differentiation, as well as the distinctness and uniformity, of 84 (88.4%) out of 95 cultivars, including 90 Korean and five Dutch cultivars. All cultivars that were not distinguished by the SNP markers were Frisée d’Amerique type. Since they were mostly developed by the same companies, their genetic similarities are probably due to the similar breeding program including the use of the same inbred line.

If a minimal amount of markers can be used for cultivar identification without decreasing resolution, the cost, time, and labor required for cultivar identification will be significantly decreased, thus increasing the overall efficiency of the method. The core sets of markers (192, 96, 48, and 24 SNPs) selected from 294 filtered SNPs, and the subsets of core markers also successfully distinguished lettuce cultivars by identifying 84 (88.4%) of the 95 studied cultivars similar to the 294 markers (Fig. 2). Therefore, the core set of 24 markers developed in this study will be a useful tool for new cultivar registration and protection by supporting the selection of the “reference varieties” for DUS testing. The additional reduced number of marker sets were analyzed to identify the minimal number of SNP markers that can distinguish the tested cultivars. Eighteen markers also identified 88.4% of the studied cultivars and they can be substituted when needed to reduce the number of markers while maintaining the identification rates of the core marker set. The additional reduced number of marker sets such as 12, nine, and six SNP markers respectively distinguished 66 (69.5%), 47 (49.5%) and 15 (15.8%) cultivars, with lower identification rates proportional to the applied marker numbers in inverse order. Therefore, the subsets of SNP markers would also be useful for quick identification of lettuce cultivars. The application of the reduced number of SNP markers will enhance the efficiency of cultivar identification at a lower cost and with reduced effort.

In this study, the grouping results based on Fluidigm genotyping data showed a similar pattern to those obtained from 17 877 SNP markers from GBS data. The 24 core SNPs classified 95 lettuce cultivars into three distinct clusters and each cluster showed a tendency to be grouped with the same horticultural type (Fig. 3). This result showed that the developed SNP markers may be effectively used for genetic diversity and marker-trait association studies of lettuce. Although there is no direct evidence that the developed markers are associated with morphological traits, further in-depth studies on associations between genetic markers and phenotypic traits would allow better identification of cultivars. A previous study reported that 384 SNPs from 298 homozygous lettuce lines were used to assess the association between SNPs and ten horticultural traits, resulting in the detection of nine significant marker-trait associations [32]. Therefore, associations between molecular markers and morphological traits will contribute to the precise cultivar identification as a supplement to morphological analysis.

Since lettuce is a principally self-pollinated crop, homozygous genotypes are predicted to be predominant. Here, at first, the results obtained from automated calling data showed an unusually high portion of heterozygous SNPs. Therefore, all 192 SNPs were reanalyzed and the automatically-called heterozygous genotypes by the software were changed to homozygote genotypes. However, heterozygous genotypes at the polymorphic loci among 192 SNPs were still observed at a low level, accounting for 0.25% of the total genotyping results. The appearance of heterozygous genotypes in lettuce cultivars could have been caused by technical limitations, incomplete fixation of cultivars, seed purity problems. Therefore, the core 24 SNPs, which showed a clear separation between two homozygous genotypes were selected for lettuce cultivar identification (Table 3, 4).

To validate that the 24 core markers accurately differentiated between homozygous and heterozygous genotypes, additional genotyping was performed (Fig. 4). As F_1_ cultivars were not available, we generated artificial heterozygous lines by mixing equal amounts of DNAs from two homozygous lines with different alleles. Of the genotyping results, 93.3% were heterozygous. This demonstrated that the developed markers could identify not only homozygous lines but also heterozygous lines.

DUS testing is required for plant cultivar registration and protection, and SNP markers have successfully supported the management of reference collections, including the selection of a “similar variety” as the test cultivar with the highest genetic similarity [7]. As mentioned above, the 24 core markers developed in this study and the total of 294 filtered SNPs that identified 89.5% of the 95 lettuce cultivars can be used to reduce higher numbers of cultivars and/or breeding lines candidates for DUS. For instance, genetically similar cultivars and/or breeding lines selected for further DUS testing can be narrowed down for morphological characterization saving resources and time to seed companies. The marker sets developed in this study can be used to select the reference varieties for use in the DUS test by screening genetically similar cultivars. Therefore, the developed markers will be valuable resources to improve the DUS system and construct a DNA-based PVP system.

In conclusion, genome-wide SNP marker discovery via GBS and SNP genotyping using the Fluidigm system was successfully applied to assess the genetic diversity of lettuce (*L. sativa*) and validate the selected core sets of markers for cultivar identification as a part of the DUS test of lettuce. Based on these results, we constructed SNP database for lettuce cultivar identification using the genotyping results of Korean commercial lettuce cultivars. The constructed SNP database will support cultivar identification, population structure analysis, lettuce breeding, and DUS test for plant cultivar protection and enforcement of the right of breeders. Further research of SNP genotyping using the core marker sets developed in this study for additional lettuce cultivars will facilitate the utility of the markers to identify diverse cultivars worldwide. Additionally, a genome-wide association study and quantitative trait locus mapping are needed to understand the association between marker and trait.

## Materials and methods

### Plant materials and DNA extraction

Ninety Korean commercial lettuce cultivars were used to obtain whole-genome data of lettuce cultivars, and five lettuce cultivars from the Netherlands were added for marker validation (Table S5). Their horticultural types were determined by the KSVS, Gimcheon, Korea, based on UPOV TG/13/10 guidelines for lettuce [4]. In this study, the Cos type was the most abundant, accounting for more than 50% of 90 cultivars, followed by the Frisée d’Amérique type and the Butterhead type. Seeds were obtained from each cultivar grown at a greenhouse in KSVS (Gimcheon, Korea), and young leaf tissue was collected from each of the 95 lettuce cultivars. Genomic DNA was extracted using a DNeasy 96 Plant kit (Qiagen, cat. no. 69181, Germany) according to the manufacturer’s instructions. DNA concentration and quality were determined using NanoDrop 8000 (Thermo Scientific, USA). The extracted DNA was normalized and used for GBS library construction and SNP genotyping. For SNP genotyping using the Fluidigm system, the concentration of DNA samples was adjusted to 10 ng/μL.

### GBS

GBS libraries were constructed for the 90 lettuce cultivars following the protocols described by Elshire et al. [69]. In brief, the genomic DNA of each cultivar was digested with *Ape*Kl (New England Biolabs, Ipswitch, MA, USA) and ligated to a specific barcode adapter. Digested DNAs were pooled and purified with the QIAquick PCR Purification Kit (Qiagen, Valencia, CA, USA).

GBS libraries were sequenced using the HiSeq2000 (Illumina, Inc., San Diego, CA, USA) by SEEDERS Inc. (Daejeon, Korea) and demultiplexing was conducted based on barcode sequence information. Adapter trimming was performed using the Cutadapt (v.1.8.3) program [70] and sequence quality trimming was conducted using the DynamicTrim and LengthSort program of the SolexaQA (v.1.13) package [71]. The quality control standards were: i) minimum phred score of 20 and ii) minimum read length of 25. The cleaned reads were aligned to the reference genome sequence of lettuce [2] using Burrows-Wheeler Aligner (BWA 0.6.1-r104) [72] with default parameters except the following options: i) seed length (−l) = 30, ii) maximum differences in the seed (−k) = 1, iii) number of threads (−t) = 32, iv) mismatch penalty (−M) = 6, v) gap open penalty (-O) = 15, and vi) gap extension penalty (−E) = 8.

### SNP calling

SNP calling was conducted by SEEDERS Inc. (Daejeon, Korea) with an in-house script [73] and SAMtools (v.0.1.16) [74] with default parameters, except the following options: i) minimum mapping quality for SNPs ≥30, ii) mapping quality for gaps ≥15, iii) read depth ≥ 3 and ≤ 190, iv) minimum InDel score for nearby SNP filtering ≥30. SNP matrix was generated and filtered with the following conditions: i) minimum depth ≥ 3, ii) MAF > 5%, and iii) missing data <30%, using in-house script [73]. The raw SNP positions identified from each sample were integrated, and the non-SNP loci were filled with the consensus sequence of the sample and the miscalled lloci were filtered out. The physical positions of the SNPs in the genome, such as coding sequence (CDS), intronic, or intergenic region, were identified.

### Genetic differentiation analysis

To estimate genetic differentiation and the number of subpopulations among lettuce cultivars using the filtered SNPs, the 95 lettuce accessions were analyzed and clustered via hierarchical clustering, PCA, and population structure analysis. Phylogenetic analysis was conducted using the NJ method implemented in Molecular Evolutionary Genetics Analysis 6 (MEGA 6) [75], and a phylogenetic tree was visualized using the bootstrap method. PCA was conducted based on 17 877 SNPs using the R package SNPRelate [76]. The population structure analysis was conducted using STRUCTURE software [77], and each number of assumed clusters (*K*) was set from 1 to 10. The optimal *K* value was calculated using the Delta-K method (Δ*K*) described by Evanno et al. [78].

To develop markers suitable for lettuce cultivar identification, we selected SNPs based on the PIC value and chromosomal position. The PIC value for SNP markers was calculated according to the following formula:

$$ \mathrm{PIC}=1-\sum_{i=1}^n{p}_i^2-\sum_{i=1}^{\mathrm{n}-1}\sum_{j=i+1}^n2{p}_i^2{p}_j^2 $$

where *n* is the number of alleles and *p_i_* and *p_j_* are the frequency of the *i*  ^th^ and *j*  ^th^ allele, respectively.

### SNP validation and genotyping

To validate SNPs discovered from GBS data, Sanger sequencing was conducted using the flanking sequences of the selected SNPs. For high-throughput analysis using the Fluidigm system, we converted SNPs from GBS into SNP type assays to be used in Fluidigm 192.24, 96.96, 48.48, or 24.192 dynamic arrays, which yielded data points with 192, 96, 48, or 24 markers, respectively. The primer sequences of the selected SNP markers are listed in Table S4. The selected SNPs were validated via a high-throughput Fluidigm Juno™ system (Fluidigm Corporation, San Francisco, CA, United States) with 95 lettuce cultivars, according to the manufacturer’s instructions. To test the efficiency of the prepared SNP assays, DNAs of five cultivars from the Netherlands were also included in the Fluidigm assay (Table S5). Sets of specific target amplification (STA) primer, a locus-specific primer (LSP), and an allele-specific primer (ASP) were designed for each SNP using the Fluidigm D3 Assay Design (https://d3.fluidigm.com/; Fluidigm, South San Francisco, CA, USA) [50]. Template sequences were prepared at a length of 200–300 bp, including 100 bp upstream and downstream of the targeted SNP. The 96.96 IFC (Fluidigm, South San Francisco, CA, USA) was used to perform the SNP type assays according to the manufacturer’s instructions [50]. The pre-amplification step was performed using LSP and STA primers and the pre-amplified products were amplified using a set of ASPs.

Further, fluorescence intensity was quantified using the Fluidigm EP1 (Fluidigm Corporation, San Francisco, CA, USA), and the SNPs were called using Fluidigm SNP Genotyping Analysis software v4.5.1 according to the manufacturer’s protocol. The allelic data matrix of “1” or “0” was used for the population genetic analysis for the genotyping results. A phylogenetic tree was generated based on SNP markers via NTSYS-pc 2.2 program (Applied Biostatistic, New York, USA) [79] using sequential agglomerative hierarchical nested clustering analysis.

To demonstrate if the developed markers worked accurately, we conducted SNP genotyping with 24 core markers, as well as 20 sample sets with two cultivars and their DNA mixture, using the Fluidigm system. The genotype results of the DNA mixtures were compared with those of the original cultivars.

## Supplementary Material

Web_Material_uhac119

## Data Availability

GBS reads of the 90 Korean lettuce cultivars have been deposited in the National Center for Biotechnology Information (NCBI) with BioProject accession number PRJNA746621 (Release data: 01-01-2022). The genotypic data of the developed SNP markers for the 90 and 5 Korean and Dutch cultivars are included as supplementary information. All relevant data of this study are included in this published article and its supplementary information files.
