# Supplementary material for: Genome-wide core sets of SNP markers and Fluidigm assays for rapid and effective genotypic identification of Korean cultivars of lettuce (Lactuca sativa L.)
Source: Hortic Res. 2022 May 26;9:uhac119. doi: 10.1093/hr/uhac119 (PMC9343917; doi:10.1093/hr/uhac119)
Supplement: Web_Material_uhac119 [file web_material_uhac119.zip › Figure S1.pptx]

## Slide 1
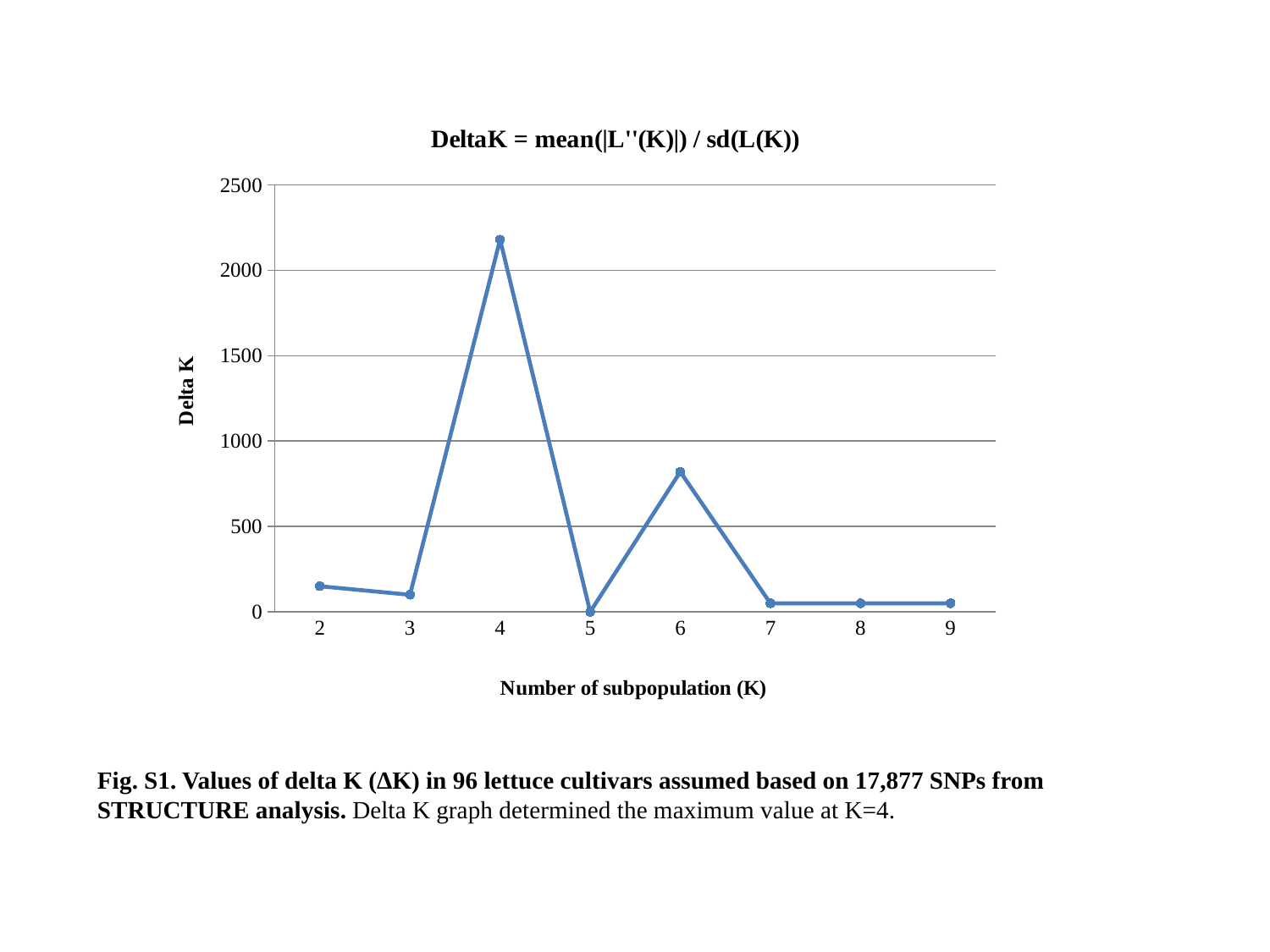

### Chart: DeltaK = mean(|L''(K)|) / sd(L(K))
| Category | Delta K |
|---|---|
| 2 | 150.0 |
| 3 | 100.0 |
| 4 | 2180.0 |
| 5 | 0.0 |
| 6 | 820.0 |
| 7 | 50.0 |
| 8 | 50.0 |
| 9 | 50.0 |Fig. S1. Values of delta K (ΔK) in 96 lettuce cultivars assumed based on 17,877 SNPs from STRUCTURE analysis. Delta K graph determined the maximum value at K=4.
