# Supplementary material for: Genome-wide core sets of SNP markers and Fluidigm assays for rapid and effective genotypic identification of Korean cultivars of lettuce (Lactuca sativa L.)
Source: Hortic Res. 2022 May 26;9:uhac119. doi: 10.1093/hr/uhac119 (PMC9343917; doi:10.1093/hr/uhac119)
Supplement: Web_Material_uhac119 [file web_material_uhac119.zip › Figure S2.pptx]

## Slide 1
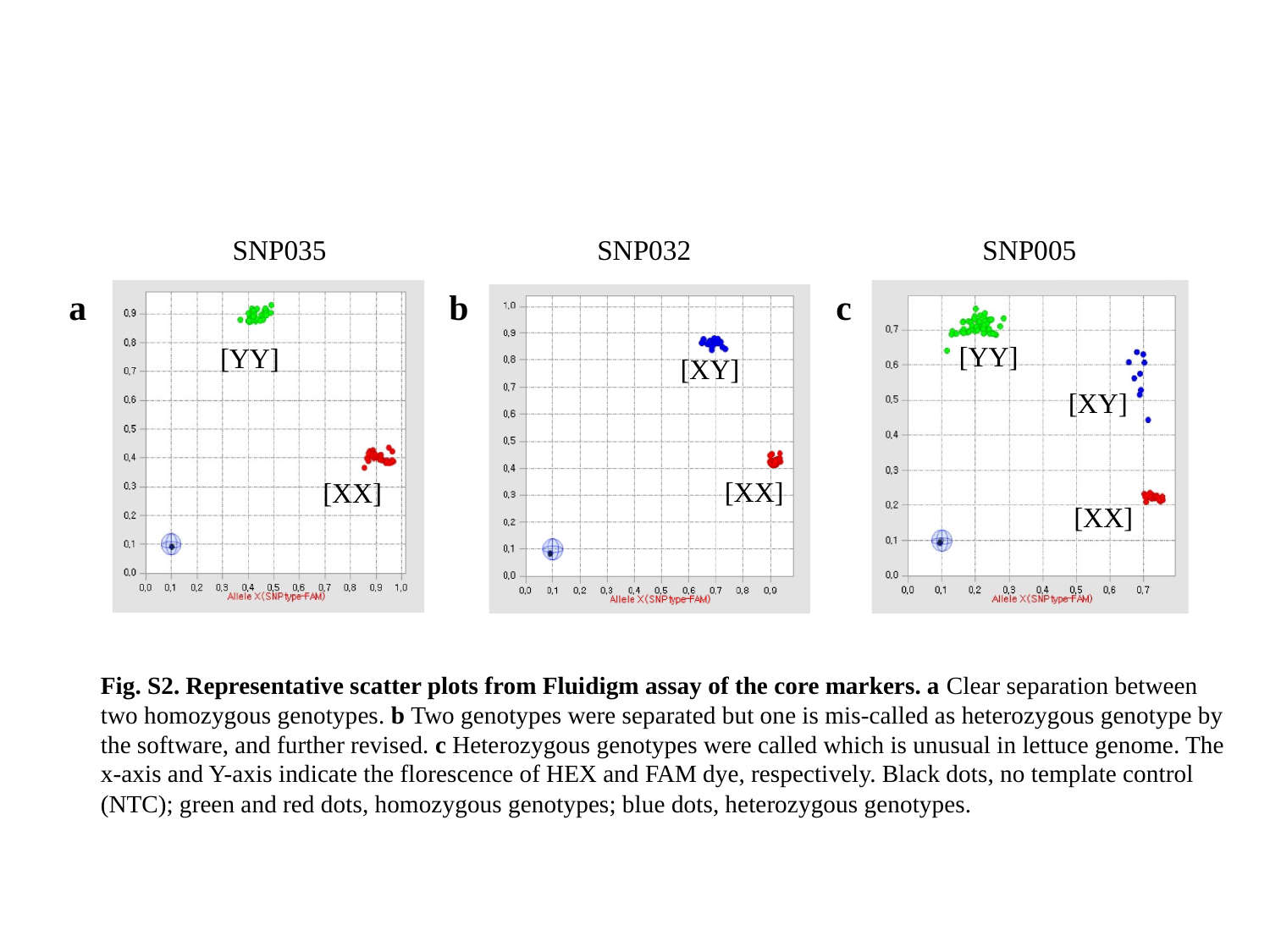

SNP032
SNP005
SNP035
a
c
b
[YY]
[YY]
[XY]
[XY]
[XX]
[XX]
[XX]
Fig. S2. Representative scatter plots from Fluidigm assay of the core markers. a Clear separation between two homozygous genotypes. b Two genotypes were separated but one is mis-called as heterozygous genotype by the software, and further revised. c Heterozygous genotypes were called which is unusual in lettuce genome. The x-axis and Y-axis indicate the florescence of HEX and FAM dye, respectively. Black dots, no template control (NTC); green and red dots, homozygous genotypes; blue dots, heterozygous genotypes.
