# Supplementary material for: Genome-wide core sets of SNP markers and Fluidigm assays for rapid and effective genotypic identification of Korean cultivars of lettuce (Lactuca sativa L.)
Source: Hortic Res. 2022 May 26;9:uhac119. doi: 10.1093/hr/uhac119 (PMC9343917; doi:10.1093/hr/uhac119)
Supplement: Web_Material_uhac119 [file web_material_uhac119.zip › Figure S3.pptx]

## Slide 1
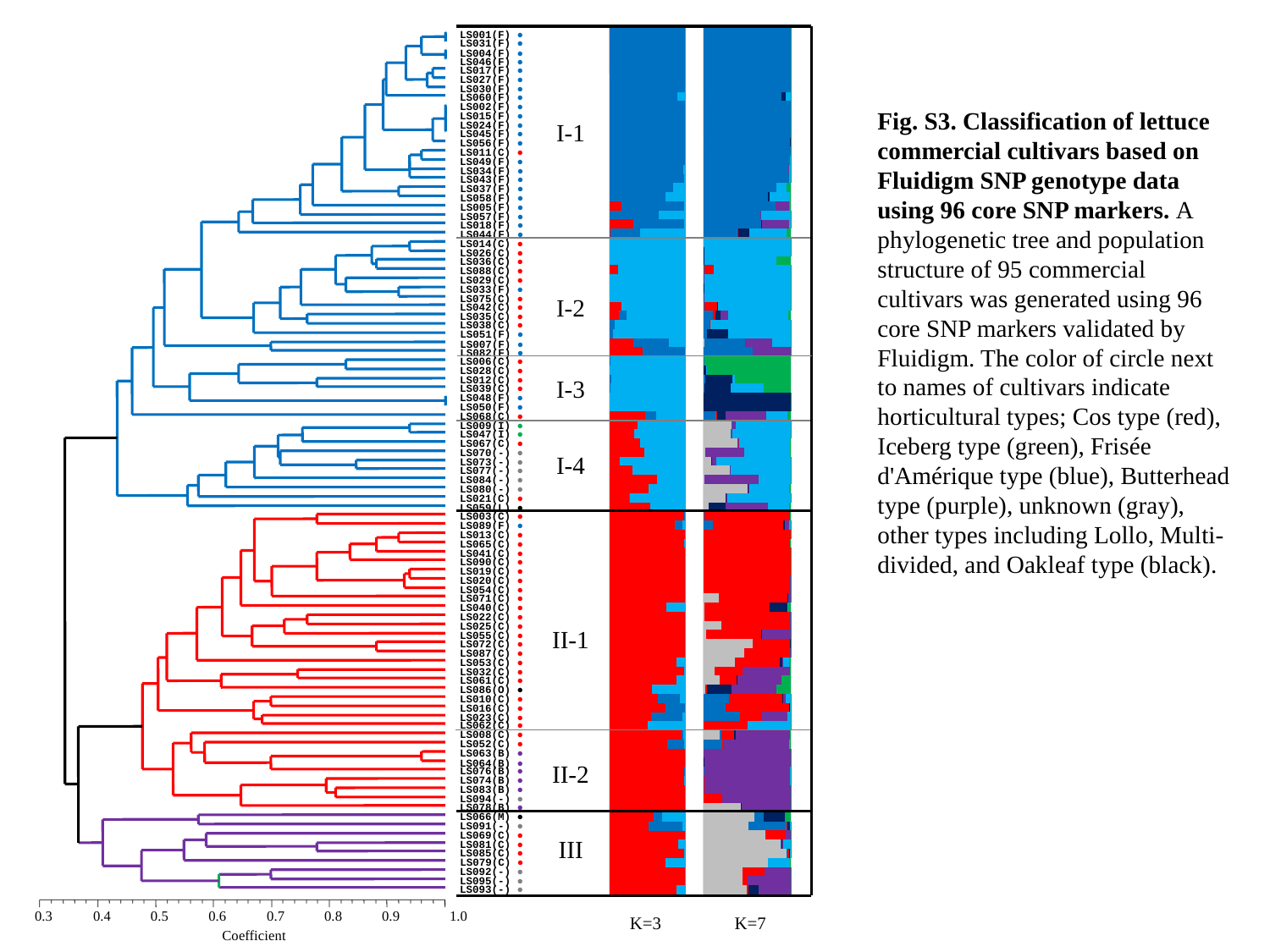

### Chart
| Category | P1 | P2 | P3 | P4 | P5 | P6 | P7 |
|---|---|---|---|---|---|---|---|
| LS093 | 0.494 | 0.004 | 0.02 | 0.11 | 0.368 | 0.001 | 0.002 |
| LS095 | 0.447 | 0.003 | 0.055 | 0.002 | 0.488 | 0.002 | 0.003 |
| LS092 | 0.447 | 0.001 | 0.254 | 0.001 | 0.295 | 0.001 | 0.001 |
| LS079 | 0.735 | 0.001 | 0.001 | 0.003 | 0.001 | 0.244 | 0.015 |
| LS085 | 0.953 | 0.006 | 0.024 | 0.003 | 0.002 | 0.012 | 0.001 |
| LS081 | 0.882 | 0.006 | 0.006 | 0.005 | 0.013 | 0.087 | 0.001 |
| LS069 | 0.71 | 0.001 | 0.232 | 0.001 | 0.054 | 0.001 | 0.001 |
| LS091 | 0.517 | 0.438 | 0.001 | 0.031 | 0.002 | 0.011 | 0.001 |
| LS066 | 0.582 | 0.108 | 0.001 | 0.241 | 0.001 | 0.002 | 0.065 |
| LS078 | 0.425 | 0.002 | 0.005 | 0.002 | 0.565 | 0.002 | 0.001 |
| LS094 | 0.003 | 0.002 | 0.211 | 0.001 | 0.78 | 0.002 | 0.001 |
| LS083 | 0.001 | 0.001 | 0.004 | 0.001 | 0.991 | 0.001 | 0.001 |
| LS074 | 0.001 | 0.001 | 0.005 | 0.001 | 0.976 | 0.015 | 0.001 |
| LS076 | 0.001 | 0.007 | 0.004 | 0.002 | 0.979 | 0.006 | 0.001 |
| LS064 | 0.001 | 0.001 | 0.003 | 0.001 | 0.991 | 0.002 | 0.001 |
| LS063 | 0.001 | 0.001 | 0.001 | 0.001 | 0.994 | 0.001 | 0.001 |
| LS052 | 0.001 | 0.215 | 0.003 | 0.004 | 0.759 | 0.003 | 0.014 |
| LS008 | 0.18 | 0.026 | 0.142 | 0.017 | 0.625 | 0.004 | 0.007 |
| LS062 | 0.001 | 0.001 | 0.491 | 0.002 | 0.01 | 0.494 | 0.001 |
| LS023 | 0.001 | 0.419 | 0.249 | 0.002 | 0.294 | 0.035 | 0.001 |
| LS016 | 0.001 | 0.254 | 0.726 | 0.004 | 0.006 | 0.007 | 0.001 |
| LS010 | 0.001 | 0.292 | 0.612 | 0.01 | 0.022 | 0.061 | 0.002 |
| LS086 | 0.019 | 0.001 | 0.024 | 0.278 | 0.514 | 0.001 | 0.163 |
| LS061 | 0.183 | 0.001 | 0.199 | 0.009 | 0.501 | 0.001 | 0.106 |
| LS032 | 0.123 | 0.001 | 0.318 | 0.008 | 0.54 | 0.002 | 0.009 |
| LS053 | 0.358 | 0.004 | 0.513 | 0.025 | 0.008 | 0.069 | 0.023 |
| LS087 | 0.461 | 0.001 | 0.529 | 0.002 | 0.005 | 0.001 | 0.001 |
| LS072 | 0.566 | 0.0 | 0.426 | 0.001 | 0.003 | 0.001 | 0.002 |
| LS055 | 0.029 | 0.0 | 0.635 | 0.001 | 0.332 | 0.001 | 0.002 |
| LS025 | 0.202 | 0.0 | 0.786 | 0.001 | 0.009 | 0.001 | 0.001 |
| LS022 | 0.009 | 0.001 | 0.982 | 0.001 | 0.006 | 0.001 | 0.001 |
| LS040 | 0.008 | 0.001 | 0.742 | 0.207 | 0.001 | 0.007 | 0.033 |
| LS071 | 0.174 | 0.001 | 0.79 | 0.001 | 0.033 | 0.001 | 0.001 |
| LS054 | 0.001 | 0.001 | 0.984 | 0.001 | 0.011 | 0.001 | 0.001 |
| LS020 | 0.001 | 0.001 | 0.991 | 0.001 | 0.005 | 0.001 | 0.001 |
| LS019 | 0.001 | 0.001 | 0.992 | 0.002 | 0.002 | 0.001 | 0.001 |
| LS090 | 0.001 | 0.001 | 0.995 | 0.001 | 0.001 | 0.001 | 0.001 |
| LS041 | 0.001 | 0.0 | 0.994 | 0.001 | 0.001 | 0.001 | 0.002 |
| LS065 | 0.001 | 0.0 | 0.984 | 0.001 | 0.001 | 0.001 | 0.013 |
| LS013 | 0.001 | 0.001 | 0.993 | 0.001 | 0.001 | 0.001 | 0.003 |
| LS089 | 0.001 | 0.104 | 0.806 | 0.024 | 0.048 | 0.012 | 0.005 |
| LS003 | 0.001 | 0.005 | 0.983 | 0.001 | 0.002 | 0.001 | 0.007 |
| LS059 | 0.055 | 0.001 | 0.003 | 0.191 | 0.483 | 0.242 | 0.025 |
| LS021 | 0.251 | 0.003 | 0.003 | 0.004 | 0.009 | 0.725 | 0.006 |
| LS080 | 0.501 | 0.003 | 0.004 | 0.003 | 0.01 | 0.462 | 0.018 |
| LS084 | 0.01 | 0.001 | 0.002 | 0.001 | 0.618 | 0.367 | 0.001 |
| LS077 | 0.298 | 0.003 | 0.001 | 0.002 | 0.004 | 0.688 | 0.003 |
| LS073 | 0.084 | 0.005 | 0.001 | 0.008 | 0.047 | 0.854 | 0.001 |
| LS070 | 0.016 | 0.001 | 0.002 | 0.003 | 0.441 | 0.528 | 0.009 |
| LS067 | 0.39 | 0.002 | 0.002 | 0.002 | 0.01 | 0.584 | 0.011 |
| LS047 | 0.314 | 0.002 | 0.001 | 0.002 | 0.014 | 0.666 | 0.002 |
| LS009 | 0.324 | 0.002 | 0.001 | 0.002 | 0.04 | 0.629 | 0.002 |
| LS068 | 0.002 | 0.147 | 0.003 | 0.097 | 0.469 | 0.246 | 0.038 |
| LS050 | 0.001 | 0.001 | 0.001 | 0.996 | 0.001 | 0.001 | 0.001 |
| LS048 | 0.001 | 0.001 | 0.001 | 0.996 | 0.001 | 0.001 | 0.001 |
| LS039 | 0.001 | 0.007 | 0.002 | 0.301 | 0.001 | 0.373 | 0.315 |
| LS012 | 0.001 | 0.021 | 0.002 | 0.304 | 0.002 | 0.03 | 0.64 |
| LS028 | 0.001 | 0.002 | 0.001 | 0.028 | 0.001 | 0.003 | 0.964 |
| LS006 | 0.001 | 0.009 | 0.001 | 0.001 | 0.001 | 0.001 | 0.986 |
| LS082 | 0.002 | 0.557 | 0.001 | 0.001 | 0.437 | 0.001 | 0.001 |
| LS007 | 0.009 | 0.47 | 0.007 | 0.002 | 0.298 | 0.212 | 0.002 |
| LS051 | 0.001 | 0.04 | 0.001 | 0.238 | 0.005 | 0.714 | 0.002 |
| LS038 | 0.001 | 0.072 | 0.001 | 0.004 | 0.001 | 0.92 | 0.001 |
| LS035 | 0.001 | 0.103 | 0.031 | 0.063 | 0.088 | 0.679 | 0.034 |
| LS042 | 0.005 | 0.001 | 0.153 | 0.003 | 0.001 | 0.834 | 0.004 |
| LS075 | 0.0 | 0.003 | 0.001 | 0.001 | 0.001 | 0.993 | 0.001 |
| LS033 | 0.001 | 0.002 | 0.001 | 0.003 | 0.001 | 0.992 | 0.001 |
| LS029 | 0.001 | 0.002 | 0.001 | 0.001 | 0.001 | 0.993 | 0.001 |
| LS088 | 0.005 | 0.002 | 0.108 | 0.001 | 0.001 | 0.88 | 0.003 |
| LS036 | 0.001 | 0.002 | 0.002 | 0.001 | 0.001 | 0.831 | 0.162 |
| LS026 | 0.001 | 0.002 | 0.001 | 0.002 | 0.001 | 0.992 | 0.002 |
| LS014 | 0.0 | 0.002 | 0.001 | 0.001 | 0.001 | 0.993 | 0.001 |
| LS044 | 0.001 | 0.387 | 0.009 | 0.13 | 0.001 | 0.424 | 0.047 |
| LS018 | 0.001 | 0.663 | 0.001 | 0.002 | 0.315 | 0.015 | 0.004 |
| LS057 | 0.002 | 0.646 | 0.001 | 0.005 | 0.001 | 0.344 | 0.001 |
| LS005 | 0.001 | 0.821 | 0.004 | 0.001 | 0.152 | 0.003 | 0.017 |
| LS058 | 0.001 | 0.735 | 0.002 | 0.019 | 0.002 | 0.231 | 0.011 |
| LS037 | 0.001 | 0.83 | 0.002 | 0.003 | 0.004 | 0.116 | 0.046 |
| LS043 | 0.001 | 0.979 | 0.001 | 0.001 | 0.001 | 0.013 | 0.004 |
| LS034 | 0.001 | 0.966 | 0.001 | 0.003 | 0.005 | 0.021 | 0.002 |
| LS049 | 0.001 | 0.99 | 0.001 | 0.001 | 0.001 | 0.006 | 0.001 |
| LS011 | 0.0 | 0.995 | 0.001 | 0.001 | 0.001 | 0.002 | 0.001 |
| LS056 | 0.001 | 0.984 | 0.001 | 0.008 | 0.002 | 0.003 | 0.001 |
| LS045 | 0.0 | 0.996 | 0.0 | 0.001 | 0.001 | 0.001 | 0.001 |
| LS024 | 0.0 | 0.996 | 0.0 | 0.001 | 0.001 | 0.001 | 0.001 |
| LS015 | 0.0 | 0.996 | 0.0 | 0.001 | 0.001 | 0.001 | 0.001 |
| LS002 | 0.0 | 0.996 | 0.0 | 0.001 | 0.001 | 0.001 | 0.001 |
| LS060 | 0.001 | 0.886 | 0.001 | 0.048 | 0.001 | 0.06 | 0.003 |
| LS030 | 0.0 | 0.996 | 0.0 | 0.001 | 0.001 | 0.001 | 0.001 |
| LS027 | 0.0 | 0.996 | 0.0 | 0.001 | 0.001 | 0.001 | 0.001 |
| LS017 | 0.0 | 0.996 | 0.0 | 0.001 | 0.0 | 0.001 | 0.001 |
| LS046 | 0.0 | 0.996 | 0.0 | 0.001 | 0.0 | 0.001 | 0.001 |
| LS004 | 0.0 | 0.996 | 0.0 | 0.001 | 0.0 | 0.001 | 0.001 |
| LS031 | 0.0 | 0.996 | 0.0 | 0.001 | 0.0 | 0.001 | 0.001 |
| LS001 | 0.0 | 0.996 | 0.0 | 0.001 | 0.0 | 0.001 | 0.001 |
### Chart
| Category | ace b dgf | | |
|---|---|---|---|
| LS093 | 0.882 | 0.004 | 0.113 |
| LS095 | 0.99 | 0.003 | 0.007 |
| LS092 | 0.996 | 0.001 | 0.003 |
| LS079 | 0.737 | 0.001 | 0.262 |
| LS085 | 0.979 | 0.006 | 0.016 |
| LS081 | 0.901 | 0.006 | 0.093 |
| LS069 | 0.996 | 0.001 | 0.003 |
| LS091 | 0.52 | 0.438 | 0.043 |
| LS066 | 0.584 | 0.108 | 0.308 |
| LS078 | 0.9949999999999999 | 0.002 | 0.005 |
| LS094 | 0.994 | 0.002 | 0.004 |
| LS083 | 0.996 | 0.001 | 0.003 |
| LS074 | 0.982 | 0.001 | 0.017 |
| LS076 | 0.984 | 0.007 | 0.009000000000000001 |
| LS064 | 0.995 | 0.001 | 0.004 |
| LS063 | 0.996 | 0.001 | 0.003 |
| LS052 | 0.763 | 0.215 | 0.021 |
| LS008 | 0.947 | 0.026 | 0.028 |
| LS062 | 0.502 | 0.001 | 0.497 |
| LS023 | 0.544 | 0.419 | 0.038000000000000006 |
| LS016 | 0.733 | 0.254 | 0.012 |
| LS010 | 0.635 | 0.292 | 0.073 |
| LS086 | 0.557 | 0.001 | 0.44200000000000006 |
| LS061 | 0.883 | 0.001 | 0.11599999999999999 |
| LS032 | 0.9810000000000001 | 0.001 | 0.019000000000000003 |
| LS053 | 0.879 | 0.004 | 0.117 |
| LS087 | 0.995 | 0.001 | 0.004 |
| LS072 | 0.995 | 0.0 | 0.004 |
| LS055 | 0.996 | 0.0 | 0.004 |
| LS025 | 0.997 | 0.0 | 0.003 |
| LS022 | 0.997 | 0.001 | 0.003 |
| LS040 | 0.751 | 0.001 | 0.247 |
| LS071 | 0.997 | 0.001 | 0.003 |
| LS054 | 0.996 | 0.001 | 0.003 |
| LS020 | 0.997 | 0.001 | 0.003 |
| LS019 | 0.995 | 0.001 | 0.004 |
| LS090 | 0.997 | 0.001 | 0.003 |
| LS041 | 0.996 | 0.0 | 0.004 |
| LS065 | 0.986 | 0.0 | 0.015 |
| LS013 | 0.995 | 0.001 | 0.005 |
| LS089 | 0.8550000000000001 | 0.104 | 0.041 |
| LS003 | 0.986 | 0.005 | 0.009000000000000001 |
| LS059 | 0.541 | 0.001 | 0.45799999999999996 |
| LS021 | 0.263 | 0.003 | 0.735 |
| LS080 | 0.515 | 0.003 | 0.48300000000000004 |
| LS084 | 0.63 | 0.001 | 0.369 |
| LS077 | 0.303 | 0.003 | 0.693 |
| LS073 | 0.132 | 0.005 | 0.863 |
| LS070 | 0.459 | 0.001 | 0.54 |
| LS067 | 0.402 | 0.002 | 0.597 |
| LS047 | 0.329 | 0.002 | 0.67 |
| LS009 | 0.365 | 0.002 | 0.633 |
| LS068 | 0.474 | 0.147 | 0.381 |
| LS050 | 0.003 | 0.001 | 0.998 |
| LS048 | 0.003 | 0.001 | 0.998 |
| LS039 | 0.004 | 0.007 | 0.989 |
| LS012 | 0.005 | 0.021 | 0.974 |
| LS028 | 0.003 | 0.002 | 0.995 |
| LS006 | 0.003 | 0.009 | 0.988 |
| LS082 | 0.44 | 0.557 | 0.003 |
| LS007 | 0.314 | 0.47 | 0.216 |
| LS051 | 0.007 | 0.04 | 0.954 |
| LS038 | 0.003 | 0.072 | 0.925 |
| LS035 | 0.12 | 0.103 | 0.776 |
| LS042 | 0.159 | 0.001 | 0.841 |
| LS075 | 0.002 | 0.003 | 0.995 |
| LS033 | 0.003 | 0.002 | 0.996 |
| LS029 | 0.003 | 0.002 | 0.995 |
| LS088 | 0.114 | 0.002 | 0.884 |
| LS036 | 0.004 | 0.002 | 0.994 |
| LS026 | 0.003 | 0.002 | 0.996 |
| LS014 | 0.002 | 0.002 | 0.995 |
| LS044 | 0.011 | 0.387 | 0.601 |
| LS018 | 0.317 | 0.663 | 0.020999999999999998 |
| LS057 | 0.004 | 0.646 | 0.35 |
| LS005 | 0.157 | 0.821 | 0.021 |
| LS058 | 0.005 | 0.735 | 0.261 |
| LS037 | 0.007 | 0.83 | 0.165 |
| LS043 | 0.003 | 0.979 | 0.018 |
| LS034 | 0.007 | 0.966 | 0.026000000000000002 |
| LS049 | 0.003 | 0.99 | 0.008 |
| LS011 | 0.002 | 0.995 | 0.004 |
| LS056 | 0.004 | 0.984 | 0.012 |
| LS045 | 0.001 | 0.996 | 0.003 |
| LS024 | 0.001 | 0.996 | 0.003 |
| LS015 | 0.001 | 0.996 | 0.003 |
| LS002 | 0.001 | 0.996 | 0.003 |
| LS060 | 0.003 | 0.886 | 0.111 |
| LS030 | 0.001 | 0.996 | 0.003 |
| LS027 | 0.001 | 0.996 | 0.003 |
| LS017 | 0.0 | 0.996 | 0.003 |
| LS046 | 0.0 | 0.996 | 0.003 |
| LS004 | 0.0 | 0.996 | 0.003 |
| LS031 | 0.0 | 0.996 | 0.003 |
| LS001 | 0.0 | 0.996 | 0.003 | LS001(F) ●
 LS031(F) ●
 LS004(F) ●
 LS046(F) ●
 LS017(F) ●
 LS027(F) ●
 LS030(F) ●
 LS060(F) ●
Fig. S3. Classification of lettuce commercial cultivars based on Fluidigm SNP genotype data using 96 core SNP markers. A phylogenetic tree and population structure of 95 commercial cultivars was generated using 96 core SNP markers validated by Fluidigm. The color of circle next to names of cultivars indicate horticultural types; Cos type (red), Iceberg type (green), Frisée d'Amérique type (blue), Butterhead type (purple), unknown (gray), other types including Lollo, Multi-divided, and Oakleaf type (black).
 LS002(F) ●
 LS015(F) ●
I-1
 LS024(F) ●
 LS045(F) ●
 LS056(F) ●
 LS011(C) ●
 LS049(F) ●
 LS034(F) ●
 LS043(F) ●
 LS037(F) ●
 LS058(F) ●
 LS005(F) ●
 LS057(F) ●
 LS018(F) ●
 LS044(F) ●
 LS014(C) ●
 LS026(C) ●
 LS036(C) ●
 LS088(C) ●
 LS029(C) ●
 LS033(F) ●
I-2
 LS075(C) ●
 LS042(C) ●
 LS035(C) ●
 LS038(C) ●
 LS051(F) ●
 LS007(F) ●
 LS082(F) ●
 LS006(C) ●
 LS028(C) ●
I-3
 LS012(C) ●
 LS039(C) ●
 LS048(F) ●
 LS050(F) ●
 LS068(C) ●
 LS009(I) ●
 LS047(I) ●
 LS067(C) ●
I-4
 LS070(-) ●
 LS073(-) ●
 LS077(-) ●
 LS084(-) ●
 LS080(-) ●
 LS021(C) ●
 LS059(L) ●
 LS003(C) ●
 LS089(F) ●
 LS013(C) ●
 LS065(C) ●
 LS041(C) ●
 LS090(C) ●
 LS019(C) ●
 LS020(C) ●
 LS054(C) ●
 LS071(C) ●
 LS040(C) ●
 LS022(C) ●
II-1
 LS025(C) ●
 LS055(C) ●
 LS072(C) ●
 LS087(C) ●
 LS053(C) ●
 LS032(C) ●
 LS061(C) ●
 LS086(O) ●
 LS010(C) ●
 LS016(C) ●
 LS023(C) ●
 LS062(C) ●
 LS008(C) ●
 LS052(C) ●
 LS063(B) ●
II-2
 LS064(B) ●
 LS076(B) ●
 LS074(B) ●
 LS083(B) ●
 LS094(-) ●
 LS078(B) ●
 LS066(M) ●
 LS091(-) ●
III
 LS069(C) ●
 LS081(C) ●
 LS085(C) ●
 LS079(C) ●
 LS092(-) ●
 LS095(-) ●
 LS093(-) ●
K=3
K=7
0.3
0.4
0.5
0.6
0.7
0.8
0.9
1.0
Coefficient
